# Supplementary material for: Ten Years (2011–2021) of the Italian Lombardy ADHD Register for the Diagnosis and Treatment of Children and Adolescents with ADHD
Source: Children (Basel). 2021 Jul 15;8(7):598. doi: 10.3390/children8070598 (PMC8304222; doi:10.3390/children8070598)
Supplement: Supplementary file 1 [file children-08-00598-s001.zip › children-1267105-supplementary.pdf]

Supplementary Material

1. Table S1–3, with Wicoxon effect and V Cramer added
2. Hosmer-Lemeshow test for Table S1 and S2

Table S1. Demographic Characteristics of the ADHD patients.

		ADHD yes	ADHD no	Total	p	Wilcoxon effect size (Z/√Z)	V Cramer
Children		4,091	1,843	5,934			
Age at diagnosis	median (q1 - q3)	9,0 (7,0 - 11,0)	9,0 (8,0 - 11,0)	9,0 (7,0 - 11,0)	<0.0001 *	4.4705/√5890=0.0582	
	media (ds)	9,2 (2,6)	9,4 (2,4)	9,2 (2,5)			
	(min - max)	(5,0 - 17,0)	(5,0 - 17,0)	(5,0 - 17,0)			
	Missing	30	14	44			
School age at diagnosis	5-11	3.264 (80.4)	1.447 (79.1)	4.711 (80.0)	0,2635		0.0146
	12-17	797 (19.6)	382 (20.9)	1.179 (20.0)			
	Missing	30	14	44			
Gender	Female	607 (14.8)	367 (19.9)	974 (16.4)	<0.0001 *		0.0634
	Male	3.484 (85.2)	1.476 (80.1)	4.960 (83.6)			
	Missing	-	-	-			
Only child	Yes	1.054 (25.8)	396 (21.5)	1.450 (24.5)	0,0004		0.0461
	No	3.028 (74.2)	1.443 (78.5)	4.471 (75.5)			
	Missing	9	4	13			
Born in Italy	Yes	3.869 (94.6)	1.774 (96.3)	5.643 (95.1)	0,0067		0.0352
	No	220 (5.4)	69 (3.7)	289 (4.9)			
	Missing	2	-	2			
Adopted	Yes	149 (3.6)	34 (1.8)	183 (3.1)	0,0002		0.0481
	No	3.938 (96.4)	1.807 (98.2)	5.745 (96.9)			
	Missing	4	2	6			
School	Primary School	3.124 (76.4)	1.371 (74.5)	4.495 (75.8)	0,1124		0.0206
	Middle/High School	964 (23.6)	469 (25.5)	1.433 (24.2)			
	Missing	3	3	6			
Support teacher	Yes	514 (12.6)	79 (4.3)	593 (10.0)	<0.0001		0.1277
	No	3.577 (87.4)	1.764 (95.7)	5.341 (90.0)			
	Missing	-	-	-			
Educational level of mother	Yes	2.313 (56.5)	1.116 (60.6)	3.429 (57.8)	0,0038		0.0376
	No	1.778 (43.5)	727 (39.4)	2.505 (42.2)			
	Missing	-	-	-			
Educational level of father	Yes	1.865 (45.6)	934 (50.7)	2.799 (47.2)	0,0003		0.0472
	No	2.226 (54.4)	909 (49.3)	3.135 (52.8)			
	Missing	-	-	-			
Mother employed	Yes	2.729 (66.7)	1.227 (66.6)	3.956 (66.7)	0,9210		0.0013
	No	1.362 (33.3)	616 (33.4)	1.978 (33.3)			
	Missing	-	-	-			
Father employed	Yes	3.411 (83.4)	1.624 (88.1)	5.035 (84.9)	<0.0001		0.0612
	No	680 (16.6)	219 (11.9)	899 (15.1)			
	Missing	-	-	-			
ADHD familiarity	Yes	831 (20.3)	193 (10.5)	1.024 (17.3)	<0.0001		0.1205
	No	3.260 (79.7)	1.650 (89.5)	4.910 (82.7)			
	Missing	-	-	-			
Psychiatric comorbidity	Yes	2.879 (70.4)	1.079 (58.5)	3.958 (66.7)	<0.0001		0.1161
	No	1.212 (29.6)	764 (41.5)	1.976 (33.3)			
	Missing	-	-	-			

Type of comorbidity (flag)	Learning disorder	1.594 (39.0)	613 (33.3)	2.207 (37.2)	<0.0001	0.0546
	Sleeping disorder	582 (14.2)	145 (7.9)	727 (12.3)	<0.0001	0.0897
	ODD	569 (13.9)	93 (5.0)	662 (11.2)	<0.0001	0.1303
	Anxiety	280 (6.8)	162 (8.8)	442 (7.4)	0,0083	0.0343
	Language disorder	287 (7.0)	88 (4.8)	375 (6.3)	0,0010	0.0426
	Tic	95 (2.3)	22 (1.2)	117 (2.0)	0,0038	0.0376
	Conduct disorder	69 (1.7)	41 (2.2)	110 (1.9)	0,1551	0.0185
	Coordination disorder	95 (2.3)	23 (1.2)	118 (2.0)	0,0061	0.0356
Chronic disease	Yes	265 (6.5)	122 (6.6)	387 (6.5)	0,8376	0.0027
	No Missing	3.826 (93.5)	1.721 (93.4)	5.547 (93.5)		
Type of chronic disease (flag)	Neurological	91 (2.2)	30 (1.6)	121 (2.0)	0,1324	0.0195
	Breathing	60 (1.5)	32 (1.7)	92 (1.6)	0,4365	0.0101
	Gastrointestinal	18 (0.4)	11 (0.6)	29 (0.5)	0,4227	0.0104

Test della bontà di adattamento di Hosmer e Lemeshow

**Chi-quadrato**	**DF**	**Pr > ChiQuadr**
**15.3161**	**8**	**0.0533**

Since $p > 0.05$ there isn’t an evidence of lack of fit.

Table S2. Clinical Characteristics of the ADHD Patients by Treatment Prescription.

		Pharmacological Treatment	Psychological Treatment	Total	p	V di Cramer
		734	3,282	4,016		
ADHD Subtype	Combined	586 (79.8)	1.899 (57.9)	2.485 (61.9)	<0.0001 *	0.1752
	Inattentive	122 (16.6)	1.096 (33.4)	1.218 (30.3)		
	Hyperactive	26 (3.5)	287 (8.7)	313 (7.8)		
	Missing	-	-	-		
QI pathologic	Yes	82 (11.4)	79 (2.4)	161 (4.1)	<0.0001 *	0.1755
	No	635 (88.6)	3.166 (97.6)	3.801 (95.9)		
	Missing	17	37	54		
CPRS-O	Pathological	384 (63.6)	1.305 (42.6)	1.689 (46.0)	<0.0001 *	0.1563
	Normal	220 (36.4)	1.761 (57.4)	1.981 (54.0)		
	Missing	130	216	346		
CTRS-O	Pathological	310 (59.0)	1.219 (41.7)	1.529 (44.3)	<0.0001 *	0.1257
	Normal	215 (41.0)	1.707 (58.3)	1.922 (55.7)		
	Missing	209	356	565		
CPRS-I	Pathological	515 (85.3)	2.176 (70.9)	2.691 (73.3)	<0.0001 *	0.1201
	Normal	89 (14.7)	892 (29.1)	981 (26.7)		
	Missing	130	214	344		
CTRS-I	Pathological	362 (69.0)	1.717 (58.6)	2.079 (60.2)	<0.0001 *	0.0756
	Normal	163 (31.0)	1.211 (41.4)	1.374 (39.8)		
	Missing	209	354	563		
CPRS-H	Pathological	477 (79.0)	1.840 (60.0)	2.317 (63.1)	<0.0001 *	0.1460
	Normal	127 (21.0)	1.228 (40.0)	1.355 (36.9)		
	Missing	130	214	344		
CTRS-H	Pathological	403 (76.6)	1.893 (64.7)	2.296 (66.5)	<0.0001 *	0.0911
	Normal	123 (23.4)	1.035 (35.3)	1.158 (33.5)		
	Missing	208	354	562		
CPRS-ADHD	Pathological	557 (92.2)	2.371 (77.3)	2.928 (79.7)	<0.0001 *	0.1378
	Normal	47 (7.8)	697 (22.7)	744 (20.3)		
	Missing	130	214	344		
CTRS-ADHD	Pathological	459 (87.3)	2.285 (78.1)	2.744 (79.5)	<0.0001 *	0.0818
	Normal	67 (12.7)	642 (21.9)	709 (20.5)		
	Missing	208	355	563		
CGI-S	5-7	516 (71.9)	597 (18.8)	1.113 (28.6)	<0.0001 *	0.4553
	1-4	202 (28.1)	2.575 (81.2)	2.777 (71.4)		
	Missing	16	110	126		
Psychiatric comorbidity	Yes	612 (83.4)	2.215 (67.5)	2.827 (70.4)	<0.0001 *	0.1345
	No	122 (16.6)	1.067 (32.5)	1.189 (29.6)		
	Missing	-	-	-		
Type of comorbidity (flag)	Learning Disorder	265 (36.1)	1.296 (39.5)	1.561 (38.9)	0,0890	0.0268
	Sleeping disorder	130 (17.7)	443 (13.5)	573 (14.3)	0.0032 *	0.0466
	ODD	209 (28.5)	356 (10.8)	565 (14.1)	<0.0001 *	0.1959
	Anxiety	70 (9.5)	208 (6.3)	278 (6.9)	0.0020 *	0.0487
	Intellectual disability	94 (12.8)	150 (4.6)	244 (6.1)	<0.0001 *	0.1332
	Mood disorder	57 (7.8)	170 (5.2)	227 (5.7)	0.0061 *	0.0433
	Language disorder	61 (8.3)	221 (6.7)	282 (7.0)	0,1307	0.0239
	Tic	40 (5.4)	54 (1.6)	94 (2.3)	<0.0001 *	0.0972
	Conduct disorder	29 (4.0)	39 (1.2)	68 (1.7)	<0.0001 *	0.0828
	Autism	60 (8.2)	65 (2.0)	125 (3.1)	<0.0001 *	0.1378
	Coordination disorder	28 (3.8)	63 (1.9)	91 (2.3)	0.0018 *	0.0492
	Other	17 (2.3)	66 (2.0)	83 (2.1)	0,5994	0.0083

Test della bontà di adattamento di Hosmer e Lemeshow		
**Chi-quadrato**	**DF**	**Pr > ChiQuadr**
7.2808	3	0.0635

Since p>0.05 there isn't an evidence of lack of fit.

Table S3. Symptoms severity perceptions by parents and teachers.

Conners' Rating Scales	Score	CPRS	CTRS	p	V	K (IC 95%)	Agreement %
Subscales		4,909	4,909				
O	Pathological	1.938 (39.5)	1.884 (38.4)	0,2637	0.01	0.32 (0.29 - 0.35)	68
	Normal	2.971 (60.5)	3.025 (61.6)				
I	Pathological	3.270 (66.6)	2.682 (54.6)	<0.0001	0.12	0.25 (0.22 - 0.28)	64
	Normal	1.639 (33.4)	2.227 (45.4)				
H	Pathological	2.646 (53.9)	2.846 (58.0)	<0.0001	0.04	0.31 (0.28 - 0.34)	66
	Normal	2.263 (46.1)	2.063 (42.0)				
ADHD Index	Pathological	3.530 (71.9)	3.478 (70.8)	0,2456	0.01	0.26 (0.23 - 0.29)	70
	Normal	1.379 (28.1)	1.431 (29.2)				
E	Pathological	1.645 (33.5)	1.942 (39.6)	<0.0001	0.06	0.26 (0.23 - 0.29)	65
	Normal	3.264 (66.5)	2.967 (60.4)				
CBCL	Score	Mother	Father	p	V	K (IC 95%)	Agreement %
		1,082	1,082				
I	Pathological	223 (20.6)	159 (14.7)	0.0003	0.08	0.64 (0.58 - 0.70)	89
	Normal	859 (79.4)	923 (85.3)				
E	Pathological	254 (23.5)	204 (18.9)	0.0085	0.06	0.69 (0.63 - 0.74)	89
	Normal	828 (76.5)	878 (81.1)				
T	Pathological	349 (32.3)	255 (23.6)	< 0.0001	0.10	0.66 (0.61 - 0.71	86
	Normal	733 (67.7)	827 (76.4)				
